# Fluorine-modified passivator for efficient vacuum-deposited pure-red perovskite light-emitting diodes

**DOI:** 10.1038/s41377-025-01740-1

**Published:** 2025-03-10

**Authors:** Nian Liu, Zhengzheng Liu, Yuanlong Huang, Peipei Du, Xiang Zhang, Yuxin Leng, Jiajun Luo, Juan Du, Jiang Tang

**Affiliations:** 1 Wuhan National Laboratory for Optoelectronics (WNLO) and School of Optical and Electronic Information, Huazhong University of Science and Technology (HUST), 1037 Luoyu Road, Wuhan, Hubei 430074 China; 2 State Key Laboratory of High Field Laser Physics and CAS Center for Excellence in Ultra-intense Laser Science, Shanghai Institute of Optics and Fine Mechanics (SIOM), Chinese Academy of Sciences (CAS), Shanghai, 201800 China; 3 School of Physics and Optoelectronic Engineering, Hangzhou Institute for Advanced Study, University of Chinese Academy of Sciences, Hangzhou, 310024 China; 4 Key Laboratory of Flexible Optoelectronic Materials and Technology (Jianghan University), Ministry of Education, Flexible Display Materials and Technology Co-Innovation Centre of Hubei Province and School of Optoelectronic Materials & Technology, Jianghan University, 8 Sanjiaohu Road, Wuhan, 430056 China

**Keywords:** Inorganic LEDs, Optics and photonics

## Abstract

Vacuum-deposited perovskite light-emitting diodes (PeLEDs) have demonstrated significant potential for high-color-gamut active-matrix displays. Despite the rapid advance of green PeLEDs, red ones remain a considerable challenge because of the inferior photophysical properties of vacuum-deposited red-light-emitting materials. Here, a rationally designed fluorine-modified phosphine oxide additive was introduced to in-situ passivate vacuum-deposited perovskites. The highly polar 2-F-TPPO incorporated perovskite films demonstrated enhanced photoluminescence quantum yield (PLQY), suppressed defects, and improved crystallinity. When implemented as active layers in PeLEDs, an external quantum efficiency (EQE) of 12.6% with an emission wavelength of 640 nm is achieved, which was 6 times higher compared to the previously reported most efficient vacuum-deposited red PeLEDs (EQE below 2%). Our findings lay the foundations for the further exploration of high-performance vacuum-deposited PeLEDs toward full-color perovskite displays.

## Abstract (web summary)

Efficient red vacuum-deposited perovskite light-emitting diodes were achieved by fluorine-modified passivator.

## Graphical abstract

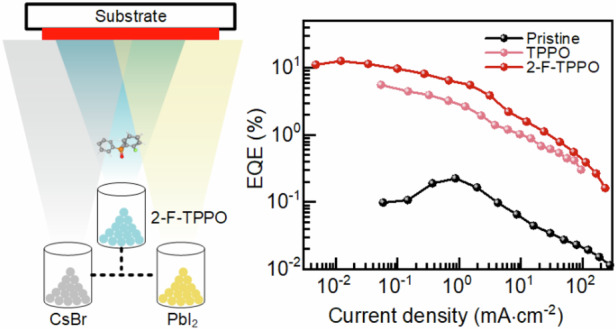

## Introduction

In recent years, perovskite light-emitting diodes (PeLEDs) have emerged as promising next-generation display technology, owing to their wide color gamut, tunable emission wavelengths, and cost-effectiveness in terms of materials and manufacturing^1–6^. Despite the significant progress achieved in external quantum efficiency (EQE) and brightness, the state-of-the-art PeLEDs are mainly fabricated by solution-processed spin coating, which is limited by laboratory-scale functional area^7–10^. Vacuum thermal deposition, a widely utilized technology in the commercial organic light-emitting diodes (OLEDs) display, offers the potential for scaling up production and ensuring reliable preparation of PeLEDs^11–14^.

Unlike traditional OLEDs, perovskites generally undergo chemical reactions in the vacuum deposition of raw materials, resulting in the preparation of perovskite films with more complex formation mechanisms. We analyze the special properties of vacuum-deposited perovskites in terms of molecular evaporation and deposition. The undesired partial decomposition of vaporized PbX_2_ molecules usually results in a greater density of halogen vacancy defects in vacuum-deposited CsPbX_3_ films which show increased trap-assisted recombination rates compared to the solution-processed perovskites^15–17^. Moreover, lead iodide is more susceptible to decomposition than lead bromide, owing to its lower bond dissociation energy^18,19^. This results in a higher density of halogen vacancy defects and inferior optoelectronic properties in vacuum-deposited iodide perovskite than in bromide perovskite. Consequently, vacuum-deposited red perovskite films often exhibit much lower PLQY than green perovskites CsPbBr_3_, thereby the best efficiency of vacuum-deposited red PeLEDs is only 1.96% so far^20–23^.

Different strategies to reduce the defect densities, including modulation of precursor ratios and reaction processes have been explored^24,25^. One of the most efficient ways is incorporating ligands which can either be in-situ added during perovskite deposition or be subsequently deposited on the perovskite film^21,26^. In addition to passivating halogen vacancy defects, such ligands can also regulate the high-energy reaction process, promoting the formation of small grains with enhanced carrier confinement^26,27^. For example, Li et al. recently reported the highest EQE of 16.4% for vacuum-deposited green PeLEDs based on co-evaporated triphenylphosphine oxide (TPPO)-CsPbBr_3_ perovskite with reduced grain size, confined charge carriers, and passivated surface defects^26^. Hsieh et al. used a guanidinium bromide (GABr) upper layer on vacuum-deposited CsPbBr_3_ perovskite for efficient passivation and preventing the undesired quenching, obtaining an EQE over 10%^21^. Although numerous approaches have been employed for defect passivation, the EQE of vacuum-deposited red PeLEDs is still limited to 1.96% and lags behind that of vacuum-deposited green PeLEDs. Manipulating electron-donating and electron-withdrawing substituents can adjust electron cloud distribution and molecular polarity, guiding an efficient passivation strategy^28–32^. Therefore, we investigate a general model leveraging electronic effects to identify suitable ligands for vacuum-deposited red perovskites exhibiting high defect densities.

In this work, we modulated the molecular properties by substituting electron-withdrawing fluorine atoms on the phenyl group of TPPO. In vacuum-deposited PeLEDs, the candidate molecules that contain π-conjugated phenyl groups are considered because of their potential for electron transfer and suitability for vacuum deposition. Among these phosphine oxides, 2-F-TPPO exhibits stronger molecular polarity and lower electrostatic potential than TPPO, resulting in 2-F-TPPO showing a better optimization effect than TPPO on the optical properties of vacuum-deposited perovskite films. Additionally, the in-situ additive 2-F-TPPO slowed the crystallization process of vacuum deposition and reduced the average grain size of perovskites from 65 nm to 45 nm. By optimizing the perovskite films, we successfully fabricated the most efficient vacuum deposited pure-red PeLED to date, achieving a record EQE of 12.6% which was 6-fold of previously reported ones. Furthermore, we demonstrated the scalability of our approach by fabricating large-area perovskite films up to 90 cm^2^. These films exhibited excellent uniformity in terms of PL emission, grain quality, and element distribution, highlighting the superior performance of the vacuum deposition method. Our research offers valuable insights into improving the performance of vacuum-deposited PeLEDs through the design of ligand molecular structures, paving the way for the commercialization of full-color PeLEDs.

## Results

### The in-situ ligand strategy in vacuum-deposited perovskites

**Fig. 1:**
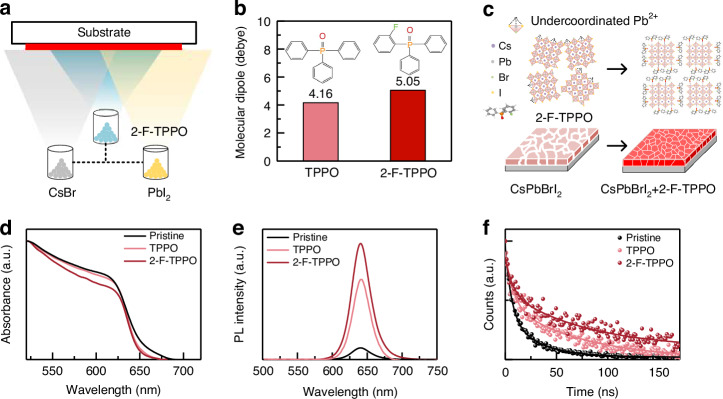
Optimization of vacuum-deposited perovskite films. **a** Schematic diagram of tri-source vacuum deposition. **b** The molecular dipole moment of TPPO and 2-F-TPPO. **c** Schematic of interaction mechanism between perovskite and 2-F-TPPO, 2-F-TPPO serves as a defect passivator and modulates the crystallization process, and 2-F-TPPO incorporated perovskite film exhibits enhanced PL emission and crystallinity. **d** UV-vis absorption, **e** PL and **f** Time-resolved photoluminescence (TRPL) spectra of films before and after the addition of phosphine oxides

The tri-source co-evaporation of CsBr, PbI_2,_ and ligand is shown schematically in Fig. 1a. Ligands have been reported to influence the perovskite films from the perspectives of film formation, passivation, and stability, thus additional requirements are claimed in ligand selection for vacuum-deposited PeLEDs. In terms of film formation, firstly, the ligand should enable controllable and stable evaporation to ensure the reliability of the deposition process. Secondly, the passivator must possess an optimal steric hindrance that is not overly large to impact the reaction process involving precursor materials. To ensure effective passivation of the defects, the P = O functional group is widely reported to strongly coordinate with Pb^2+^, therefore phosphine oxides are applied here as ligands. In this work, stabilized evaporable phosphine oxides with appropriate steric hindrance, specifically TPPO and its fluorinated derivatives are investigated as in-situ ligands. These phosphine oxides’ molecular structures, polarity, and electrostatic potentials (ESP) are shown in Fig. 1b and Fig. S1. The introduced fluorine atom around the P = O moiety (ortho, meta, and para on the phenyl group) modulated the molecular polarity and electrostatic potential of these phosphine oxides through the inductive and conjugated effects. Due to the inductive effect, the calculated permanent dipole moment of 2-F-TPPO (5.05 Debye) is larger than that of TPPO (4.16 Debye) while the dipole moments of both 3-F-TPPO and 4-F-TPPO are smaller than those of TPPO. This change in the molecular polarity stems from the inductive effect weakened with the distance between the electron-withdrawing F atom and the P = O moiety. The weaker dipole moments of 3-F-TPPO and 4-F-TPPO than that of TPPO are primarily caused by the diminished inductive effect of the F atoms on the O atoms in these molecules. Additionally, the electron-withdrawing F atoms are located farther away from the O atoms in the two phosphine oxides shown in Fig. S1b, which reduces the separation of positive and negative charges. As a result, these two phosphine oxides exhibit weaker dipole moments compared to TPPO.

The enhanced molecular polarity in 2-F-TPPO favors the interaction between the electron-donating group and the undercoordinated Pb^2+^ ions and enhances the passivation effect. Besides, the P = O moiety acts as an electronegative group in these phosphine oxides, attracting the electron cloud and leading to the distribution of negative charge (dark red region in Fig. S1) in the ESP of these molecules. The conjugation effect between the fluorine atom and the phenyl group results in electron delocalization towards the electronegative P = O group. Consequently, the electrostatic potentials on the P = O moiety vary among these phosphine oxides as follows: *φ*_min, 2-F-TPPO_<*φ*_min, TPPO_<*φ*_min, 4-F-TPPO_<*φ*_min, 3-F-TPPO_. The change in the electrostatic potential highlights the highest electron cloud density near the oxygen atom in 2-F-TPPO. Such characteristic is also expected to enhance the coordination interaction between the passivator and the undercoordinated Pb^2+^ ions, thereby providing a more effective suppression of trap-induced nonradiative recombination, as demonstrated in Fig. 1c.

The absorption and photoluminescence (PL) measurements of the pristine, TPPO, and 2-F-TPPO-incorporated perovskite films were performed. As shown in Fig. 1d, the same absorption band edges at ~636 nm of these perovskite films indicate that the additives did not influence perovskite composition. In accordance with the absorption curves, the PL emission peaks of the perovskite films are located at almost the same position while exhibiting different PL intensities in Fig. 1e. On introducing additives, the PL intensity is greatly enhanced resulting from the passivation effect of P = O moiety in vacuum-deposited perovskite film. The PL intensity is further enhanced in 2-F-TPPO incorporated perovskite film indicating better defect passivation of 2-F-TPPO which originates from the introduction of the F atom affecting the interaction between P = O moiety and perovskites^30,33,34^.

Specifically, the PLQY of TPPO and 2-F-TPPO incorporated perovskite films increased to 23.3% and 50.5% compared to the pristine film of 3.26% (Fig. S2). The TRPL measurements were carried out to investigate the carrier combination in Fig. 1f. The TRPL spectra were fitted according to a biexponential decay equation of *I*(*t*) = *I*_0_+*A*_1_exp(−*t*/*τ*_1_) +*A*_2_exp(−*t*/*τ*_2_)^35^. Average lifetimes were calculated by the equation *τ*_ave_ = (*A*_1_*τ*_1_^2^ + *A*_2_*τ*_2_^2^)/(*A*_1_*τ*_1_ + *A*_2_*τ*_2_), fitted results and the average PL lifetime *τ*_ave_ are present in Supplementary Table S1^10,36^. The *τ*_ave_ was improved from 23.61 ns (for the pristine film) to 66.42 ns (for the TPPO incorporated film) and 73.81 ns (for the 2-F-TPPO incorporated film). Thus the 2-F-TPPO incorporated film presents the longest average PL lifetime, also reflecting the differences in the passivation capabilities of the additives. Using the measured PLQYs and the fitted average PL lifetimes, the recombination rate constants were obtained according to the expression of PLQY = *k*_r_/(*k*_r_+*k*_nr_) and *τ*_ave_ = (*k*_r_+*k*_nr_)^−1^, *k*_r_ and *k*_nr_ respectively referred to radiative and non-radiative recombination rate constants (Fig. S3). Given that the *k*_nr_ decreases considerably and *k*_r_ increases, it implies that these additives effectively suppress non-radiative recombination. This also suggests that 2-F-TPPO exhibits a superior passivation effect compared to TPPO, consistent with the strong molecular polarity of 2-F-TPPO and the higher electron cloud density in the P = O group calculated before.

### The effect of ligand passivation on the carrier recombination dynamics and chemical states

**Fig. 2:**
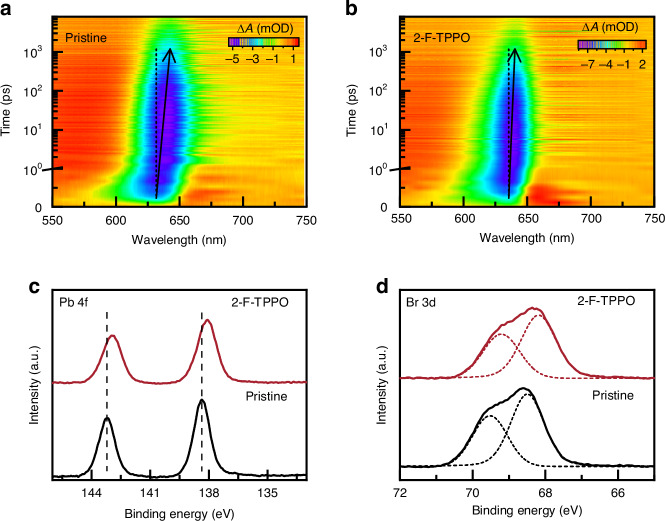
Interaction between 2-F-TPPO and perovskites. **a**, **b** Pseudo-color transient absorption spectra of the pristine film (**a**) and 2-F-TPPO incorporated perovskite film (**b**), showing the change in absorbance ($\Delta$*A*) over time. **c**, **d** XPS spectroscopy analysis of Pb 4 f (**c**) and Br 3 d (**d**) signals in the pristine and 2-F-TPPO incorporated perovskite film

Transient absorption (TA) spectroscopy measurements were further conducted to investigate the carrier dynamics in perovskite films without and with 2-F-TPPO (Fig. 2a, b). The TA results revealed a single ground state bleaching (GSB) signal peak in both cases, indicating that the addition of 2-F-TPPO did not alter the phase composition of the perovskite or affect the photophysical processes within the film. The minimum position of the GSB signal in the TA spectrum exhibits a redshift with delay time, which is related to the energy landscape and carrier relaxation in the perovskite (Fig. S4). Upon the addition of 2-F-TPPO, this redshift decreased from 27 meV to 19 meV, suggesting that 2-F-TPPO passivation resulted in a narrower energy landscape and lower density of tail state defects in the perovskite films^22,26,37^. These findings confirm the effective inhibition of 2-F-TPPO on tail states, thereby reducing non-radiative recombination in the perovskite film. We carried out X-ray photoelectron spectroscopy (XPS) to characterize the interaction between 2-F-TPPO and CsPbBrI_2_ (Fig. 2c, d). Compared to the pristine film, both the Pb 4 f peak and the Br 3 d peak shift to lower binding energy after introducing 2-F-TPPO. The reduced binding energy of the Pb 4 f orbital arises from the passivation of the undercoordinated Pb^2+^ ion by the P = O group, which in turn reduces the binding energy of the Br 3 d orbital^3,38^.

### Grain confinement passivation mechanism

**Fig. 3:**
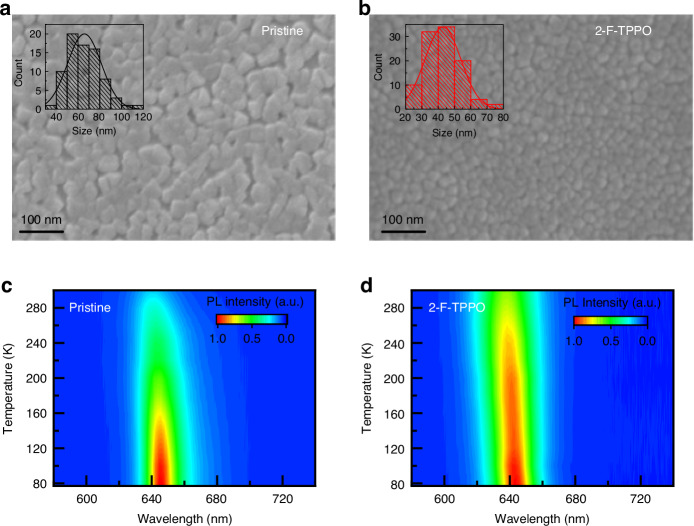
Modulate the crystallization process via 2-F-TPPO additive and suppress the tail states. **a**, **b** Top-view SEM images with grain size distribution in the insets of the pristine film (**a**) and 2-F-TPPO incorporated perovskite film (**b**). **c**, **d** Temperature-dependent PL spectra of the pristine film (**c**) and 2-F-TPPO incorporated perovskite film (**d**)

The surface morphology and grain size of perovskite films without and with 2-F-TPPO were also investigated. The SEM images of the perovskite films are shown in Fig. 3a, b, and the corresponding statistical grain size distributions are diagramed in the insets, respectively. During vacuum deposition, the high-energy, rapid, and uncontrolled crystallization process results in large grain sizes, ultimately reducing radiative recombination rates^39,40^. Therefore, the pristine perovskite film exhibits a wide grain size distribution ranging from 40–120 nm and low coverage on the substrate. The grain size of perovskite film appears to be reduced by the addition of 2-F-TPPO, decreasing from an average value of 60 nm–40 nm for the pristine and 2-F-TPPO incorporated films, respectively, and the coverage on the substrate improves with the absence of pinhole. The reduced grain size arises from the modulated vacuum deposition process by 2-F-TPPO addition and the spatial confinement of the perovskite grains, which is more conducive to radiative recombination, corresponding to the increased *k*_r_ described above^26^.

The temperature-dependent PL spectra were acquired at temperatures ranging from 80 K to 300 K to evaluate the superiority of the 2-F-TPPO incorporated films as shown in Fig. 3c, d. The location of PL peaks of both films exhibits a blue shift with the increasing temperature, which has been previously attributed to the increased bandgap caused by the expanded lattice^41–43^. The PL intensity of 2-F-TPPO incorporated film keeps relatively weakened thermal quenching across the temperature range, exhibiting superior optical characteristics (Fig. S5). The exciton binding energy (*E*_b_), which is closely associated with defect levels in the perovskite, could be calculated by fitting the integrated PL intensities *I*_PL_(T) with temperature, according to the following equation^44,45^:

$${I}_{PL}(T)=\frac{{I}_{PL}(0)}{1+A{{\rm{e}}}^{-{E}_{{\rm{b}}}/{k}_{B}T}}$$

In the equation, ${I}_{{PL}}(0)$ is the integrated PL intensity at 0 K, *k*_B_ is the Boltzmann constant and *A* is the frequency factor related to the trap states^22,45^. The calculated *E*_b_ of the pristine and 2-F-TPPO incorporated films was 47 and 161 meV, respectively. The addition of 2-F-TPPO effectively passivates the defects in perovskite film and reduces the grain size, thus greatly enhancing its exciton binding energy with a more robust exciton for strong emission.

### Device structure and performance

**Fig. 4:**
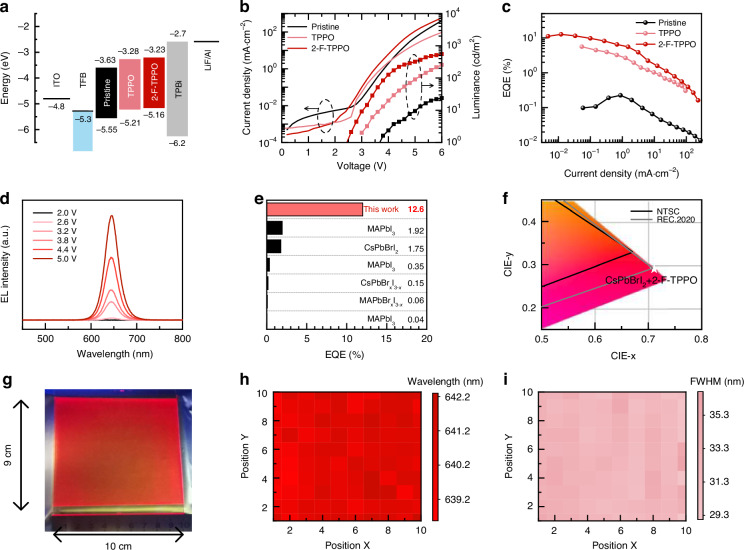
Performance of vacuum-deposited PeLEDs and large area perovskite films. **a** Energy levels of the PeLEDs. **b** Current density (*J*) and luminance (*L*) versus voltage (*V*) curves of the PeLEDs. **c** EQE versus current density curves of the PeLEDs. **d** EL spectra of the 2-F-TPPO incorporated PeLED. **e** Summary of EQE values reported for vacuum deposited red PeLEDs. **f** Comparison of the CIE coordinates of EL for the 2-F-TPPO incorporated PeLED with the NTSC and Rec. 2020 standards. **g** PL image of the 90 cm^2^ perovskite film fabricated by vacuum deposition. **h**, **i** Distribution of PL emission wavelengths (**h**) and FWHM distribution (**i**) of the large area perovskite film, each test point is >0.8 cm apart

Motivated by the excellent optoelectronic properties of 2-F-TPPO incorporated perovskite films, we further evaluated their device performance. The device structure consisted of patterned indium tin oxide (ITO)/Poly[(9,9-dioctylfluorenyl-2,7-diyl)-co-(4,4-(N-(4-sec-bu-tylphenyl)diphenylamine)] (TFB) /perovskite/1,3,5-tri(N-phenylbenzimidazol-2-yl)benzene (TPBi) /LiF/Al as depicted in Fig. 4a. We have calculated the optical band gap and carried out the ultraviolet photoelectron spectroscopy (UPS) analysis of the perovskite films, and the results are shown in Fig. S6. The energy level for the 2-F-TPPO incorporated film is upshifted compared with those of the pristine and TPPO incorporated films, suggesting a smaller hole injection barrier for the 2-F-TPPO incorporated film. TFB and TPBi were utilized as the hole and electron transportation layers, respectively. The current density-voltage-luminance (*J*−*V*−*L*) curves (Fig. 4b) revealed that the pristine perovskite-based device exhibited the lowest brightness and highest leakage current, and the maximum EQE for the pristine device was only 0.3% (Fig. 4c). This can be attributed to poor PL performance resulting from trap-assisted non-radiative recombination and inadequate surface grain coverage of the pristine film. As mentioned above, the 2-F-TPPO incorporation led to a reduction in defect density and improved grain coverage, thus corresponding devices exhibited decreased leakage current and enhanced brightness. The TPPO and 2-F-TPPO incorporated devices demonstrated maximum EQEs of 5.7% and 12.6%, respectively. The improved EQEs illustrate the effective passivation of defects by these phosphine oxides, and 2-F-TPPO performs better than TPPO. Notably, the 2-F-TPPO incorporated device exhibited significantly enhanced device performance and a substantial reduction in current density to achieve the maximum EQE. This suggests increased radiative recombination under low injected carrier concentration, attributed to the reduced crystal size after 2-F-TPPO incorporation^36,46–48^. Figure 4d illustrates the electroluminescence (EL) spectrum of the 2-F-TPPO incorporated PeLED under various bias driving voltages. The EL peak remained stable at ~640 nm without any significant shift as the driving voltage increased from 3 V to 5 V, indicating excellent spectral stability. The variation of the CIE coordinates with operating time at a driving voltage of 4.5 V was further measured to illustrate the phase stability of the CsPbBrI_2_ films (Fig. S7). The impressive EQE of 12.6% of the 2-F-TPPO incorporated device surpassed the performance of all previously reported vacuum-deposited red PeLEDs (Fig. 4e)^49–53^, bringing a five-fold improvement. Additionally, the EL spectrum of the 2-F-TPPO incorporated device achieved a CIE coordinate of (0.710,0.289), exceeding the NTSC standard red color coordinates of (0.67,0.33) and approaching the Rec. 2020 specified red color coordinates of (0.708, 0.292)^54^ as shown in Fig. 4f. The operational stability for unencapsulated pristine, TPPO and 2-F-TPPO incorporated PeLEDs is further investigated inside a nitrogen-filled glove box at 25 °C, as shown in Fig. S8. Due to the different performance parameters of the devices, the pristine, TPPO, and 2-F-TPPO devices were tested under the current density of 30 mA•cm^‐2^. The time *T*_50_ for these devices to decay to half of the initial brightness was 9.26 minutes, 37.38 minutes, and 178.38 minutes, separately. We also recorded two accelerated 100-fold videos in which the operating 2-F-TPPO incorporated PeLED operated at 4.5 V for half an hour as shown in Movie S1 and S2, Supporting Information.

Compared with the widely used solution-processed method for preparing perovskite layers, vacuum deposition offers several advantages in terms of its suitability for preparing large-area perovskite film and compatibility with current commercial OLED production lines. Consequently, the feasibility and uniformity of preparing large-area perovskite layers through vacuum deposition were verified following the successful preparation of a high-performance red PeLED. Building upon the optimized tri-source co-evaporation process, a rotary evaporation technique was employed to prepare a 9 × 10 cm^2^ 2-F-TPPO incorporated film, as depicted in Fig. 4g, supporting the feasibility of large-area perovskite preparation via vacuum deposition. Random micro-PL tests were conducted on the CsPbBrI_2_ + 2-F-TPPO film to assess the uniformity of PL emission. Utilizing a 3-μm diameter laser spot as the excitation light source in a Raman spectrometer, the PL distribution across the large-area perovskite film was examined. PL spectra were collected from 100 random points on the film, and the resulting PL emission peak and FWHM were plotted and presented in Fig. 4h, i, respectively. The test results indicate that the PL peaks of the perovskite film are distributed between 642 ~ 638 nm, with a fluctuation of 36 ~ 29 nm in the FWHM. This limited fluctuation in the CIE coordinates movement suggests that the preparation of perovskite films through vacuum deposition exhibits excellent large-area fluorescence uniformity and demonstrates the feasibility of large-area LED device fabrication.

To assess the morphology and component uniformity of the prepared large-area perovskite film, SEM and energy-dispersive spectroscopy (EDS) tests were conducted, revealing consistent grain distribution and similar grain sizes among the four groups of perovskites in Fig. S9 and Fig. S10. The EDS area scanning analysis illustrates the distribution of elements, revealing a consistent P/Pb ratio among the four randomly selected groups. This uniformity highlights the high quality of the perovskite components achieved during large-area vacuum deposition manufacturing. The exceptional uniformity of vapor-deposited perovskite films can be attributed to the significant volatility of PbI_2_, which boasts the highest vapor pressure among lead halides.

Despite the relatively short research time and few published studies, inspiring advancements have been achieved in the vacuum-deposited PeLEDs. Up to this work, the EQEs of vacuum-deposited green and red PeLEDs have exceeded 10%, with the EQE of vacuum-deposited blue PeLEDs approaching 5%. Leveraging the advantages of vacuum deposition technology in terms of pixelated deposition, it is promising to achieve full-color perovskite active-matrix display through vacuum-deposited PeLEDs. Consequently, directing efforts toward enhancing the efficiency of vacuum-deposited PeLEDs by incorporating tailored ligands emerges as a crucial research avenue for the practical implementation of PeLED display technology.

## Discussion

In summary, we demonstrate the ligand design strategy to passivate defects and boost carrier radiative recombination in vacuum-deposited perovskites to enable efficient vacuum-deposited pure red PeLEDs. First, we use theoretical calculations and experimental results to demonstrate that ortho substitution of the F atom on the phenyl group enhances the coordination ability of the P = O moiety with undercoordinated Pb^2+^ ions through electronic effects. Then, the crystallization modulation effect of 2-F-TPPO on vacuum-deposited perovskites is also revealed. Ultimately, the 2-F-TPPO incorporated device achieved a record EQE of 12.6%, far surpassing the previously reported highest efficiency of vacuum-deposited red PeLEDs by six-fold. The high uniformity of the vacuum-deposited 90 cm^2^ perovskite film was also verified, satisfying the need for large-area scalability in the display industry. This work provides an important reference for efficiency improvement in vacuum-deposited perovskites and a basis for the full-color commercial display of PeLEDs.

## Materials and methods

### Materials

Indium tin oxides (ITO) and lead iodide (PbI_2_, 5 times purified) were purchased from Advanced Election Technology Co., Ltd. Cesium bromide (CsBr, 99.99%), triphenylphosphine oxide (TPPO, 98%) and lithium fluoride (LiF, 99.99%) were purchased from Aladdin Reagent Ltd. 1-diphenylphosphoryl-2-fluorobenzene phosphine oxide (2-F-TPPO, 96%) was custom synthesized from Chemany, Inc. Poly[(9,9-dioctylfluorenyl-2,7-diyl)-alt-(4,4’-(N-(4-butylphenyl) (TFB, 4 times purified) and 1,3,5-Tris(1-phenyl-1H-benzimidazol-2-yl) benzene (TPBi) were purchased from Xi’an Yuri Solar Co., Ltd. Chlorobenzene (99.8%, super dry) was purchased from J&K Scientific Technology Co., Ltd. Aluminum pellet (Al, 99.999%) was purchased from Zhongnuo Advanced Material Technology Co., Ltd. All the materials are used as received and without further purification.

### Perovskite film deposition

The raw materials, including CsBr, PbI_2_, and phosphine oxide additives (TPPO or 2-F-TPPO), were carefully added to the quartz crucibles. The amount added accounted for approximately one-third of the crucible’s volume. The quartz crucibles were placed in folded metal molybdenum boats, ensuring close contact between the two. Inside the evaporation chamber, the molybdenum boat was positioned between the ends of the metal column. By applying current or voltage to both ends of the metal column, the raw materials inside the crucible were heated and evaporated under a high vacuum of 10^−4^ Pa. The evaporation rates were closely monitored using quartz crystal microbalance with corrected scale factors. The evaporation process was regulated by adjusting the current of the evaporation source, allowing for the production of perovskite films with varying CsBr/PbI_2_ or PbI_2_/2-F-TPPO ratios. To decelerate the reaction and crystallization kinetics during thermal evaporation, an initial evaporation rate of 0.1 Å/s was established for both CsBr and PbI_2_. It was observed that the morphology of the perovskite films was influenced by the doping ratios of CsBr/PbI_2_ and 2-F-TPPO (Fig. S11 and Fig. S12). By fine-tuning and optimizing the CsBr/PbI_2_ ratio as well as the 2-F-TPPO doping ratio, the optimal evaporation rates for CsBr, PbI_2_, and 2-F-TPPO were determined to be 0.1 Å/s, 0.1 Å/s, and 0.04 Å/s, respectively. The films were further optimized with a suitable post-annealing treatment to ensure the best fluorescence intensity and crystalline quality as shown in Figs. S13–15. The post-annealing usually leads to grain growth which in turn reduces *k*_r_, and excessive post-annealing temperatures have been proven to be harmful to the PLQY of perovskites, and therefore the PL intensities should decrease as the post-annealing temperature increases. However, the 70 °C post-annealing-treated perovskite film showed enhanced PL intensity than the 50 °C post-annealing-treated, which we attribute to the removal of non-ideal phases and the non-serve grain growth. According to Fig. S15a, the decreased XRD diffraction peaks of the 70 °C post-annealing-treated perovskite films indicate that the grain orientation became uniform and the non-ideal phases were removed, which allowed the perovskite films to exhibit the best PL performance. Consequently, in the fabrication of subsequent LED devices, post-annealing of the perovskite film was implemented to optimize its performance.

### Perovskite LED fabrication

The patterned ITO glasses were meticulously cleaned using surfactant, deionized water, and alcohol with ultrasonication. Subsequently, they were dried using a flow of dry air and treated with a plasma cleaner for 3 min. The cleaned ITO glasses were then transferred to an N_2_ glove box. A solution of TFB (4 mg/mL in chlorobenzene) was spin-coated onto the ITO substrates at 3000 rpm for 40 s and annealed at 150 °C for 20 min. After cooling, TFB substrates were transferred into the evaporation chamber for sequential deposition of perovskite (40 nm), TPBi (40 nm), LiF (1 nm), and Al (80 nm) through different shadow masks at a high vacuum of <10^−4^ Pa. The functional area of the LED device was 0.04 cm^2^ as defined by the overlapping area of patterned ITO and Al electrodes.

### Perovskite film and device characterizations

The absorption spectra were measured using a Shimadzu Solidspec-3700 spectrophotometer, while the PL spectra were recorded using a Hitachi F-7000 fluorescence spectrometer. The absolute PLQYs were determined using a Zolix OmniFluo spectrofluorometer with a calibrated integrating sphere from Labsphere. The PL lifetime was measured using an Edinburgh Instruments Ltd EPL-370. X-ray photoelectron spectroscopy (XPS) and ultraviolet photoelectron spectroscopy (UPS) were analyzed using an AXIS-ULTRA DLD-600W photoelectron spectrometer from Shimadzu-Kratos, Japan. The morphology of the perovskite films was examined using a GeminiSEM 300 scanning electron microscope (SEM) from Carl Zeiss AG. Transient absorption (TA) spectroscopy experiments were performed using a commercial Helios-EOS Ultrafast system. Temperature-dependent and spatially resolved PL spectra were obtained using a laser confocal Raman spectrometer (LabRAM HR800, Horiba JobinYvon) equipped with a temperature-controlled sample stage (LTS420, Linkan) and XYZ high-precision automatic sample stage. The characterization of the PeLED devices was carried out in the N_2_ glove box. The current density versus voltage, luminance versus voltage, and EQE versus current density curves were simultaneously recorded using a commercial measurement system (XPQY-EQE, Guangzhou Xi Pu Optoelectronics Technology) equipped with an integrating sphere. This measurement system had been calibrated by halogen lamps metered by the National Institute of Standards and Technology (NIST).

## Supplementary information

Fluorine-modified passivator for efficient vacuum-deposited pure-red perovskite light-emitting diodes

Movies S1

Movies S1

## Data Availability

The data that support the findings of this study are available from the corresponding author, upon reasonable request.
