# Supplementary material for: Fluorine-modified passivator for efficient vacuum-deposited pure-red perovskite light-emitting diodes
Source: Light Sci Appl. 2025 Mar 10;14:118. doi: 10.1038/s41377-025-01740-1 (PMC11891322; doi:10.1038/s41377-025-01740-1)
Supplement: Supplementary file 1 — Fluorine-modified passivator for efficient vacuum-deposited pure-red perovskite light-emitting diodes [file 41377_2025_1740_MOESM1_ESM.docx]

**Supplementary Information for F****luorine-modified phosphine oxide passivator for efficient vacuum-deposited pure-red perovskite light-emitting diodes**

*Nian Liu^1^*, *Zhengzheng Liu^2,3*^*, *Yuanlong Huang^1^*, *Peipei Du^4^*, *Xiang Zhang^1^*, *Yuxin Leng^2,3^, Jiajin Luo^1*^*, *Juan Du^,3*^**，Jiang Tang^1^*

^1^Wuhan National Laboratory for Optoelectronics (WNLO) and School of Optical and Electronic Information, Huazhong University of Science and Technology (HUST), 1037 Luoyu Road, Wuhan, Hubei, 430074, China.

^2^State Key Laboratory of High Field Laser Physics and CAS Center for Excellence in Ultra-intense Laser Science, Shanghai Institute of Optics and Fine Mechanics (SIOM), Chinese Academy of Sciences (CAS), Shanghai, 201800, China.

^3^School of Physics and Optoelectronic Engineering, Hangzhou Institute for Advanced Study, University of Chinese Academy of Sciences, Hangzhou, 310024, China.

^4^Key Laboratory of Flexible Optoelectronic Materials and Technology (Jianghan University), Ministry of Education, Flexible Display Materials and Technology Co-Innovation Centre of Hubei Province and School of Optoelectronic Materials & Technology, Jianghan University, 8 Sanjiaohu Road, Wuhan 430056, China.

E-mail: [liuzhengzheng@siom.ac.cn](mailto:liuzhengzheng@siom.ac.cn), luojiajun@hust.edu.cn, [du@ucas.ac.cn](mailto:du@ucas.ac.cn)


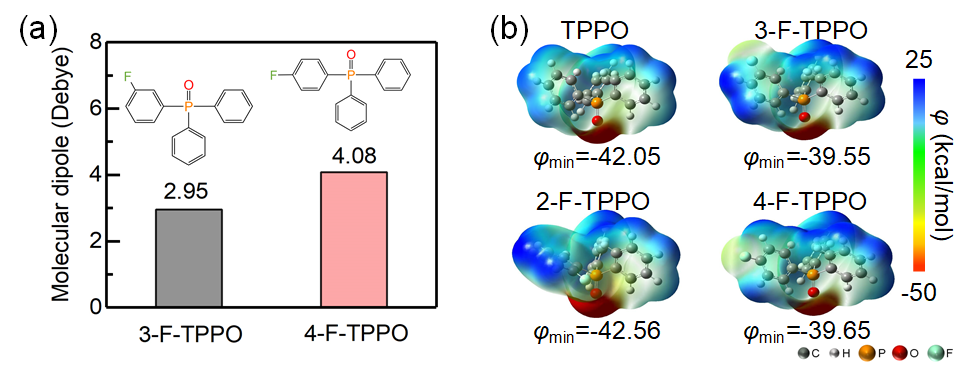
Supplementary Figures and Tables

Fig. S1 (a) The molecular dipole moment of 3-FTPPO and 4-F-TPPO. (b) The molecular structures of the phosphine oxides (TPPO, 2-FTPPO,3-F-TPPO and 4-F-TPPO). Corresponding Gaussian calculated electrostatic potentials (*φ*) are shown. The right color bar from red to blue marks the increase of electropositivity.


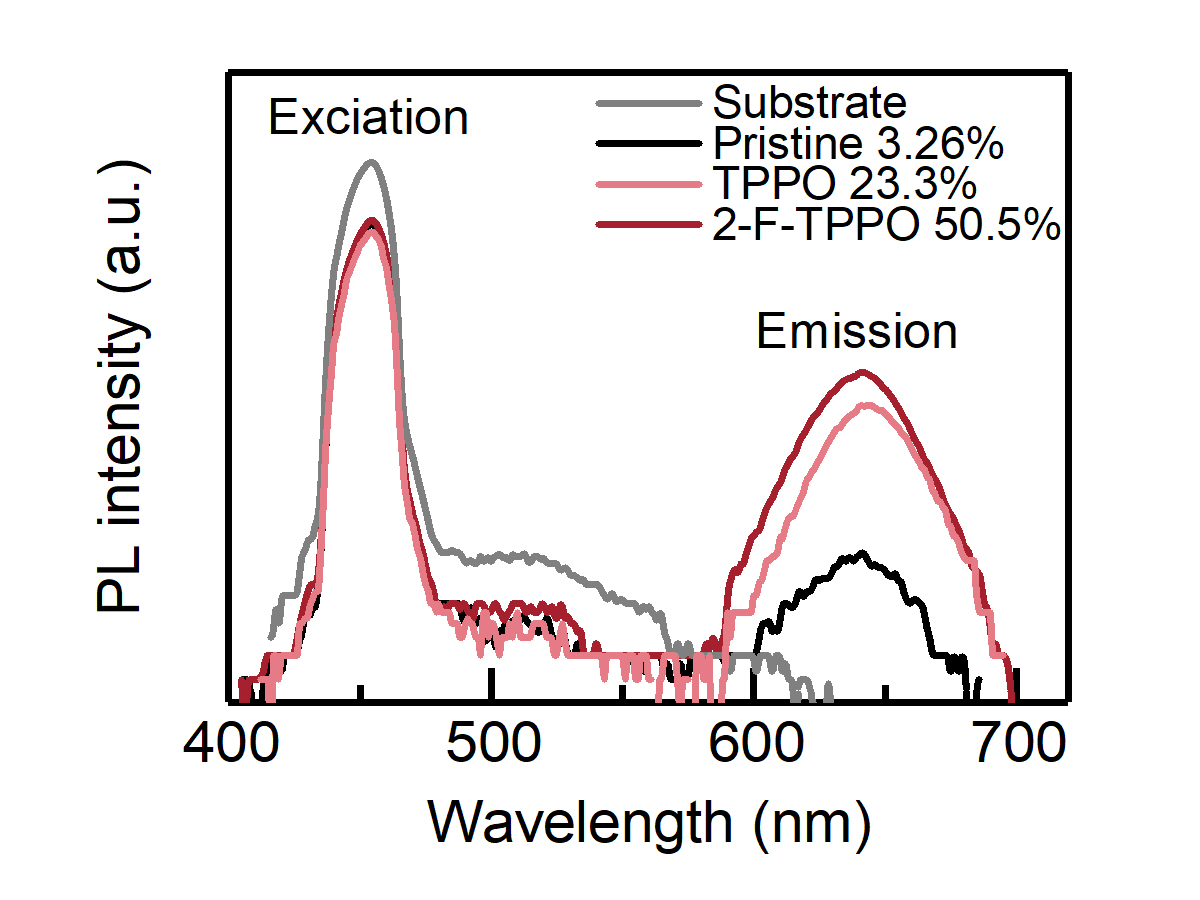
Fig. S2 PLQY of the pristine, TPPO, and 2-F-TPPO incorporated perovskite films.


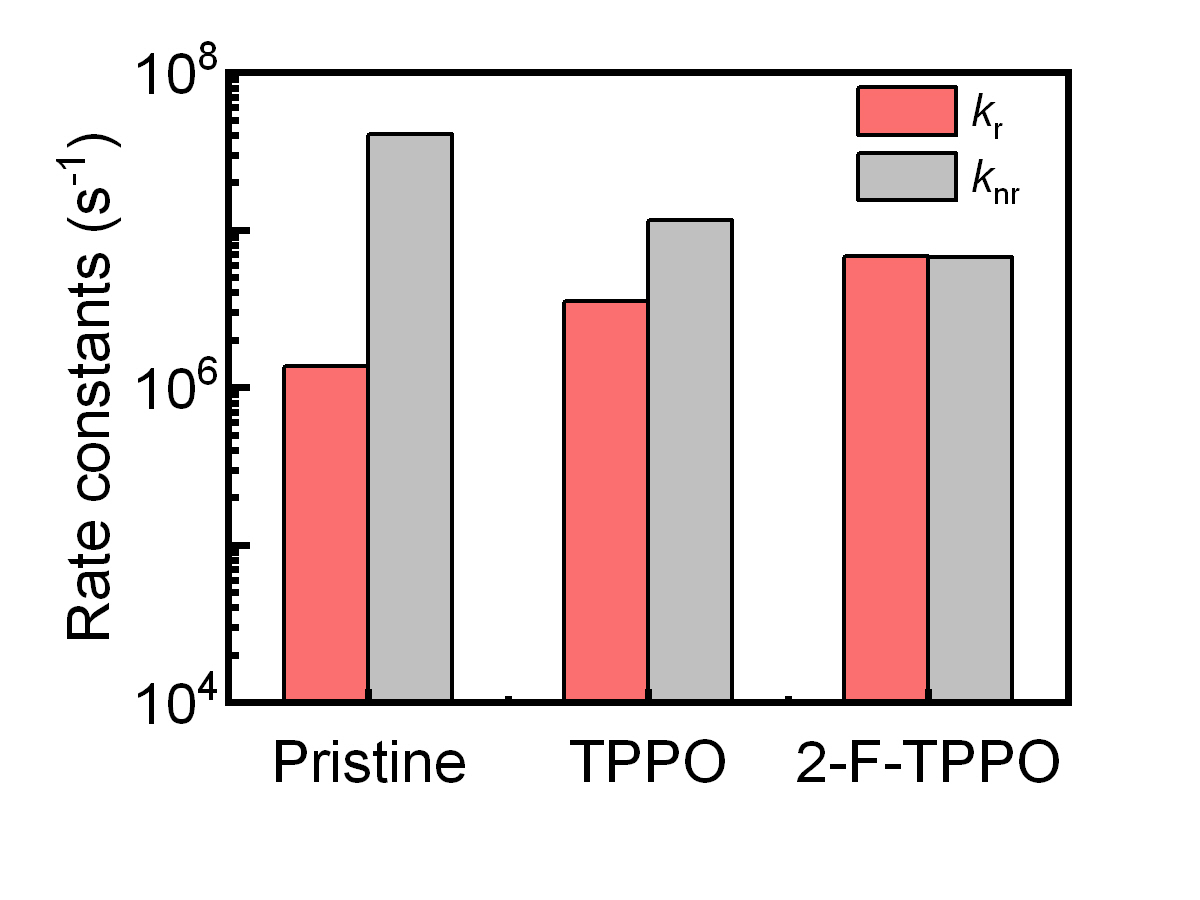
Fig. S3 Recombination rates constants *k*_r_ and *k*_nr_ of the pristine, TPPO, and 2-F-TPPO incorporated perovskite films.


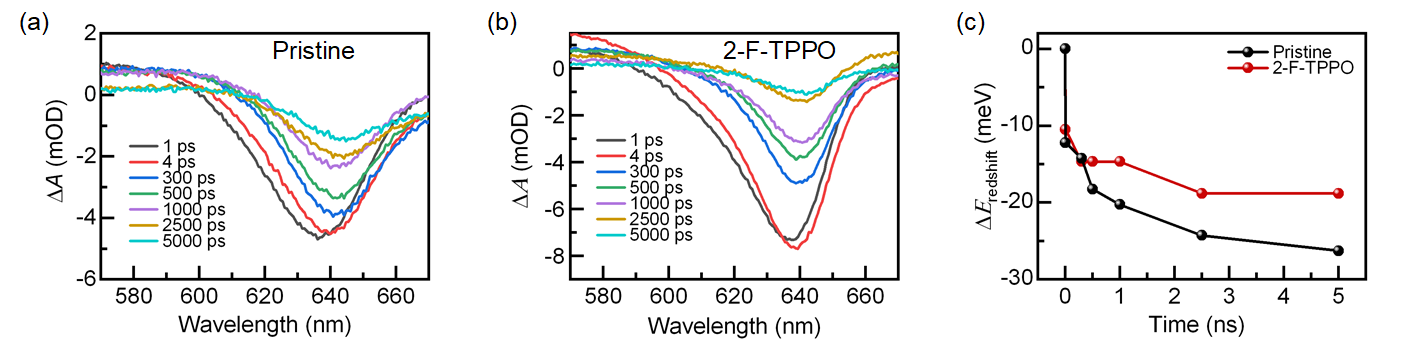
Fig. S4 The transient absorption spectra of (a) the pristine and (b) 2-F-TPPO incorporated films. (c) The shifts of the GSB peak wavelength over delay time.


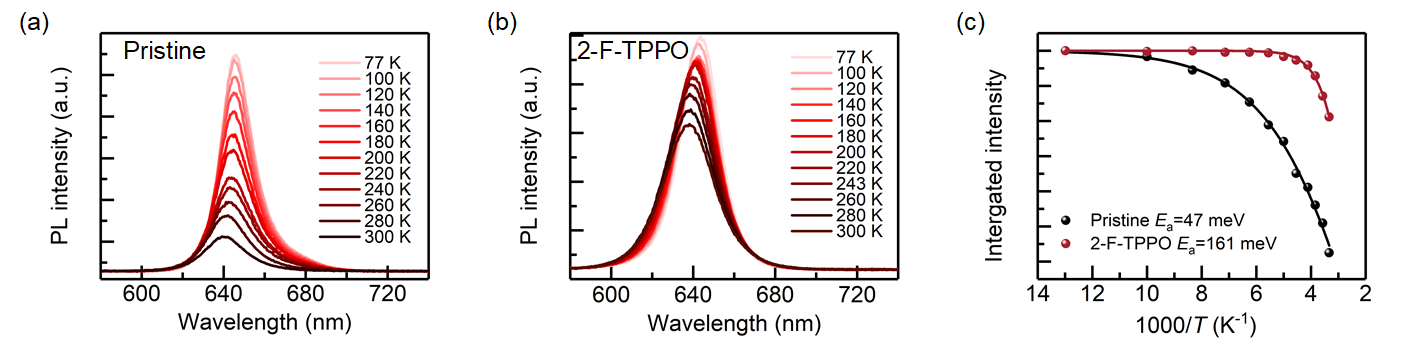
Fig. S5 PL spectra of (a) the pristine and (b) 2-F-TPPO incorporated films at increasing temperatures from 77 K to 300 K. (c) Relevant integration of the temperature-dependent PL intensity of the films and fitting curves for *E*_a_.


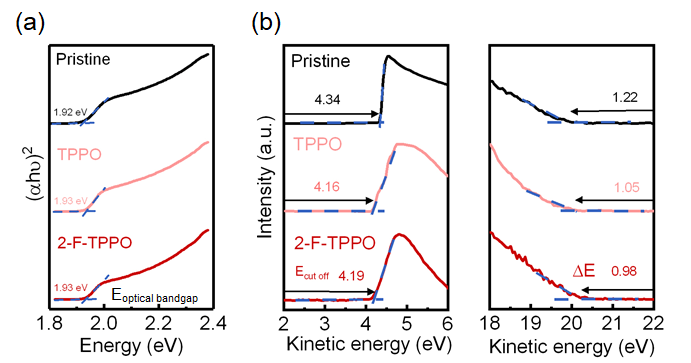
Fig. S6 (a) The plots of (*α*h*ν*)^2^ versus the photon energy calculated from the absorption measurement. (b) UPS spectra of the pristine, TPPO and 2-F-TPPO incorporated perovskite films: Photoemission cutoff region (left) and the valence-band-edge region (right). *E*_VB_ and *E*_CB_ are calculated with the formula: *E*_VB_ = *E*_cutoff_ + Δ*E*; *E*_CB_ = *E*_VB_ – *E*_optical bandgap_.


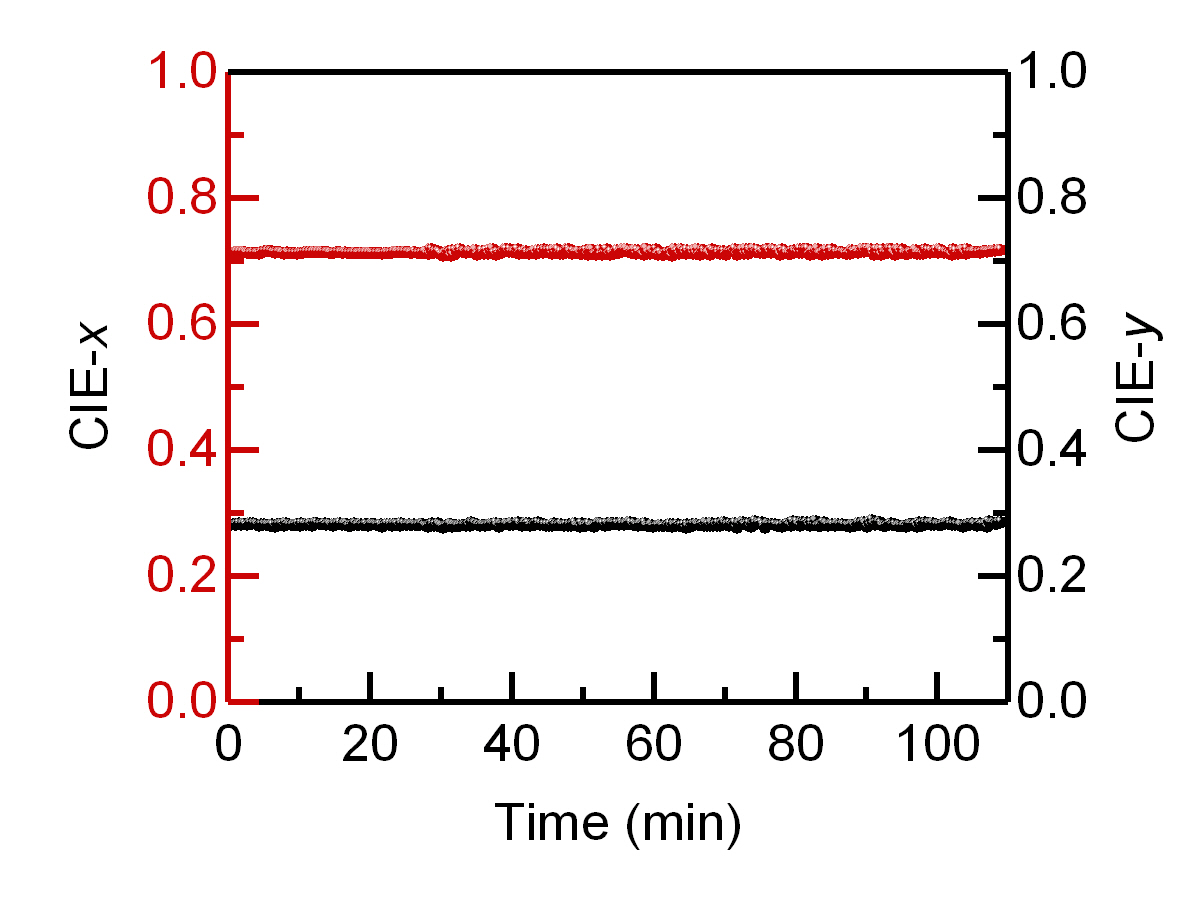
Fig. S7 The variation of the CIE coordinates with operating time at a driving voltage of 4.5 V.


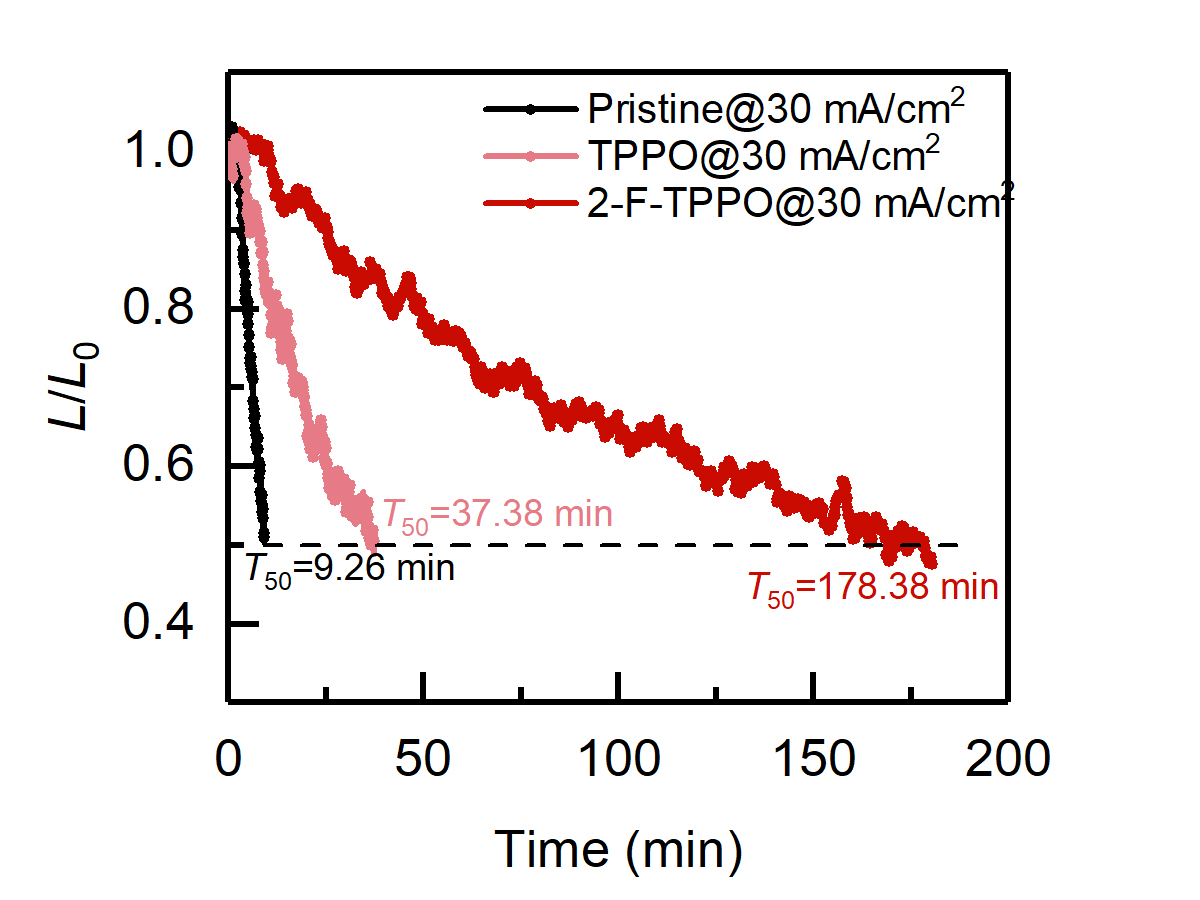
Fig. S8 The operational stability tests for the PeLEDs.


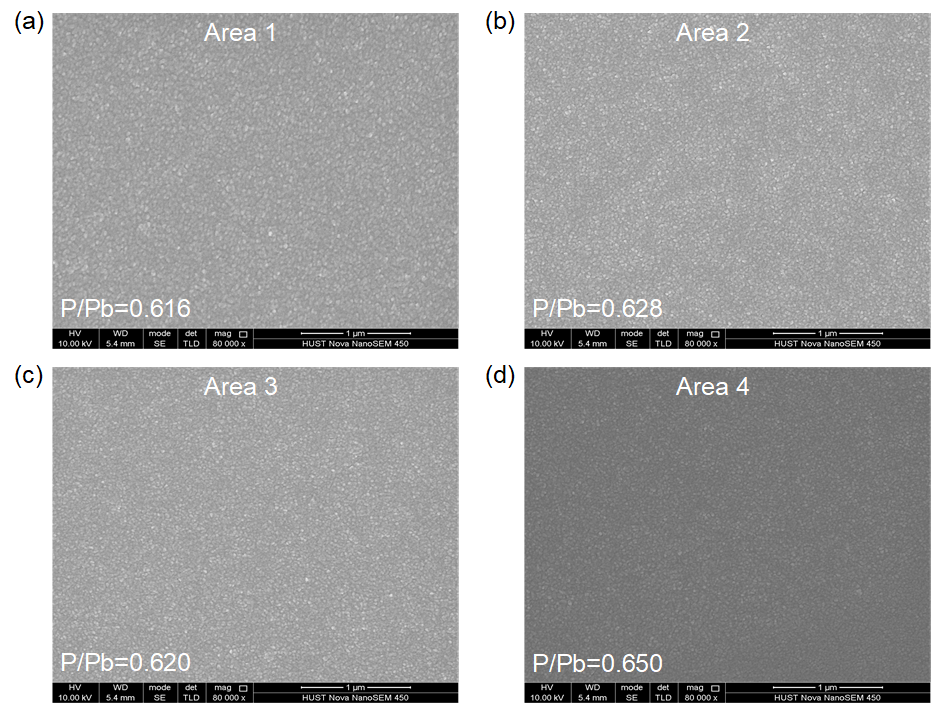
Fig. S9 Top-view SEM images of the four random areas on the large-area 90 cm^2^ perovskite film, the ratios of the elements measured by EDS are labeled in the images.


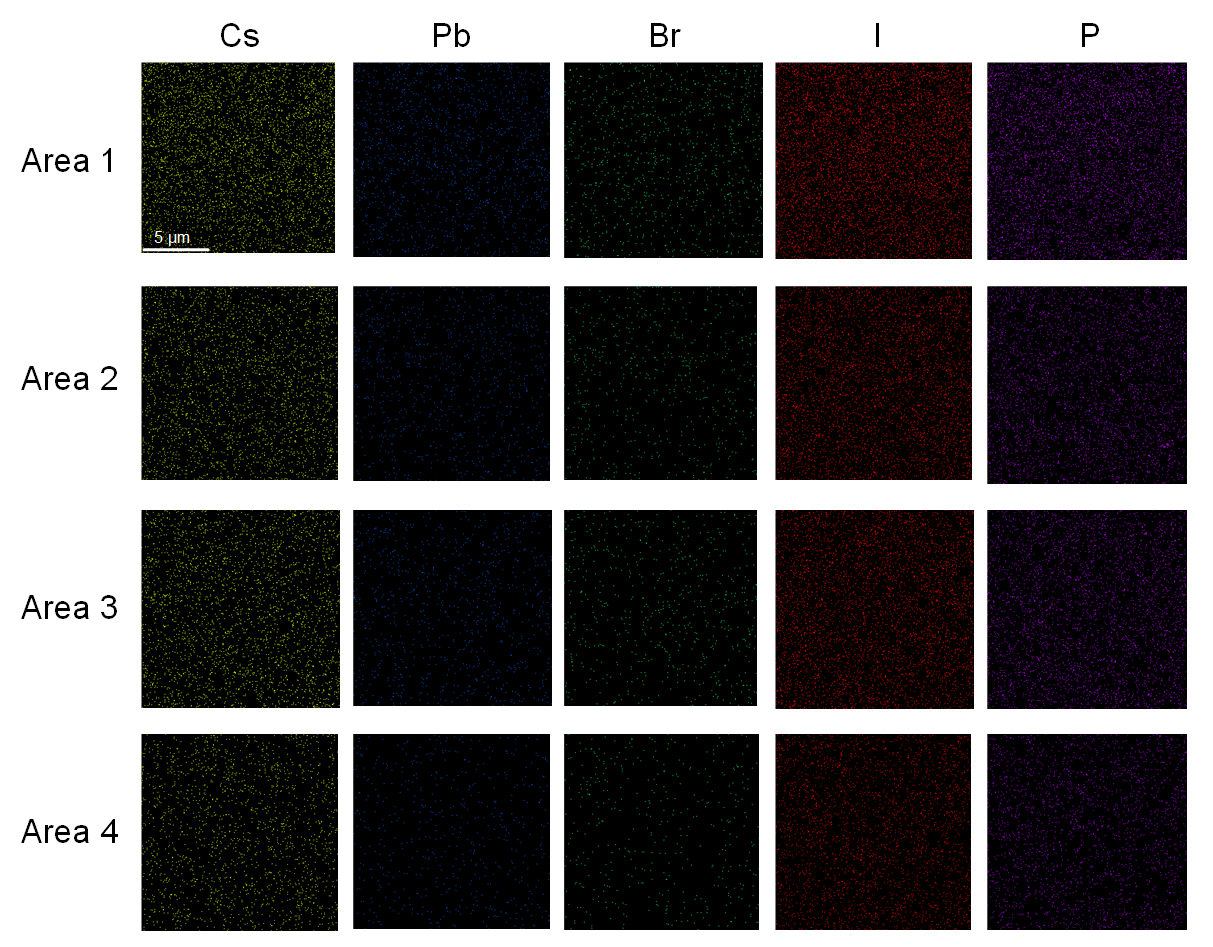
Fig. S10 The elements mapping of the four random areas on the large-area 90 cm^2^ perovskite film.


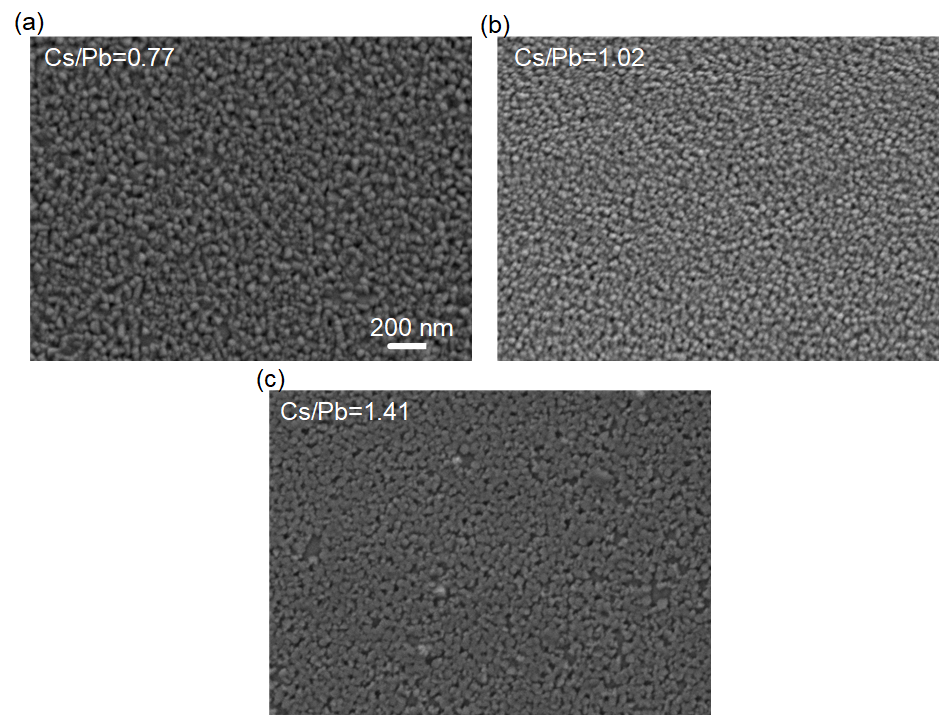
Fig. S11 SEM images of the perovskite films with different CsBr/PbI_2_ ratios, the Cs/Pb ratio measured by EDS was used as the CsBr/PbI_2_ ratio.


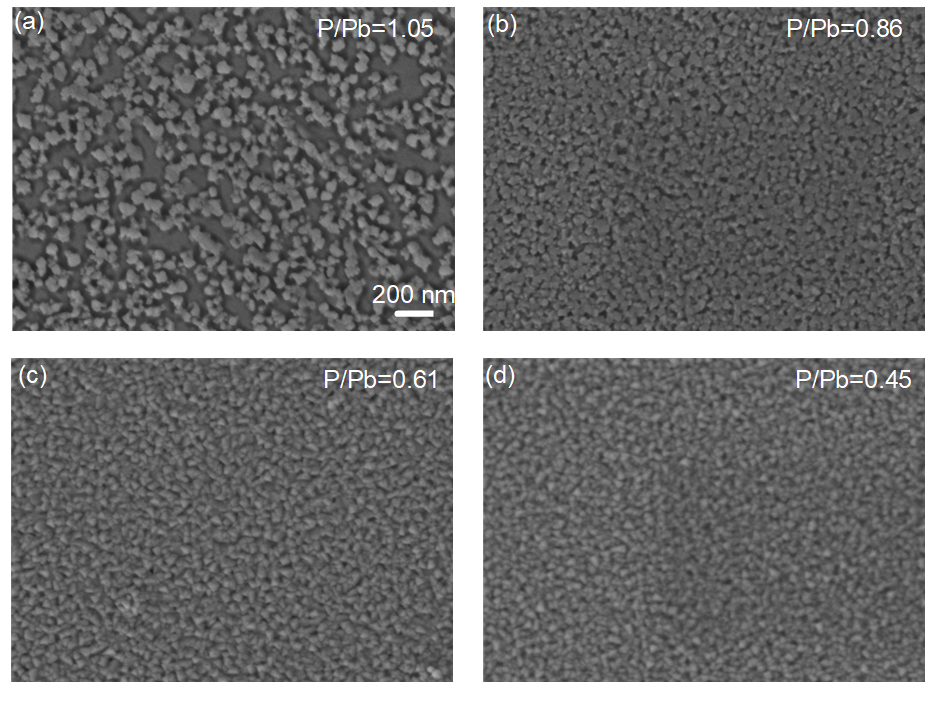
Fig. S12 SEM images of the perovskite films with different 2-F-TPPO incorporation ratios, the P/Pb ratio measured by EDS was used as the 2-F-TPPO incorporation ratio.


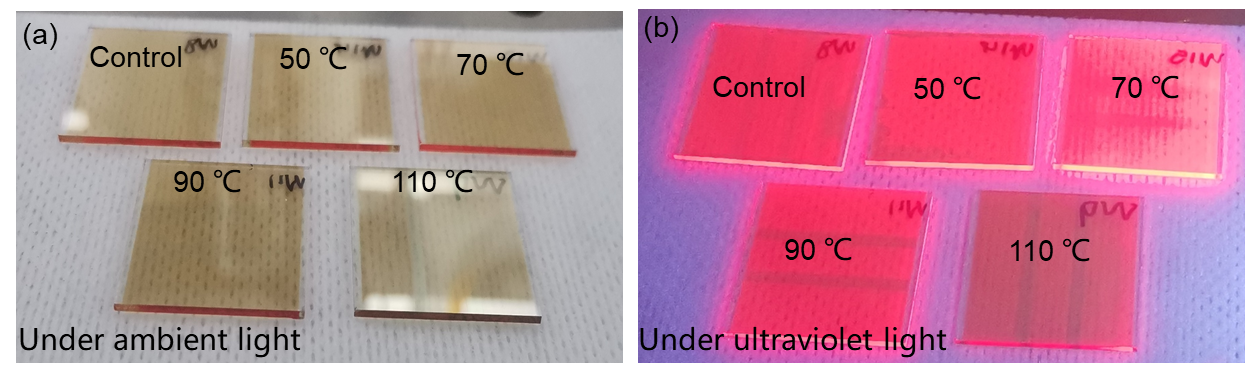
Fig. S13 Photographs of perovskite films annealed at different temperatures (a) under ambient light and (b) under UV illumination.


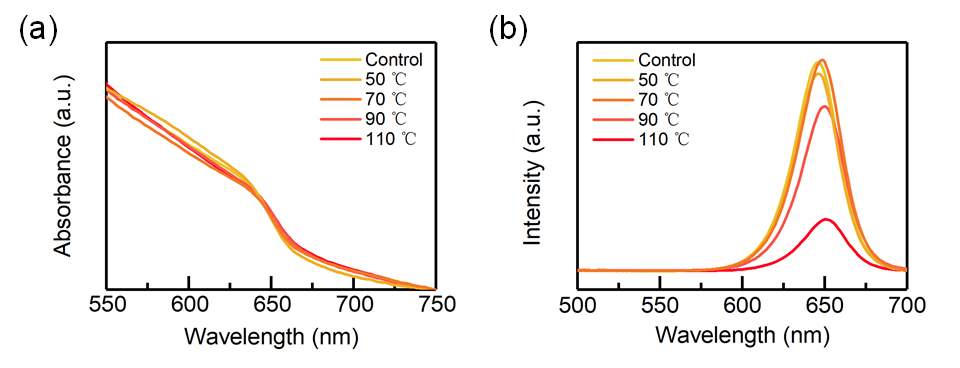
Fig. S14 (a) Absorption spectra and (b) PL spectra of perovskites annealed at various temperatures.


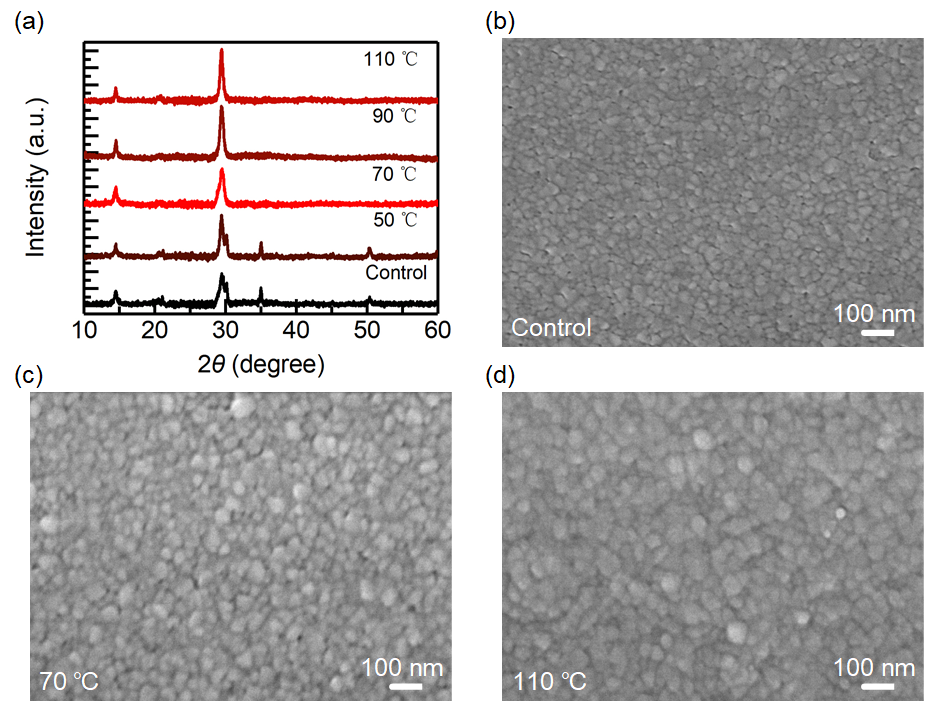
Fig. S15 (a) XRD patterns and (b-d) SEM images for 2-F-TPPO incorporated films annealed at different temperatures.

Table S1. The fitting results of TRPL transients are shown in Fig. 1f.

|  | *A*_1_ | *τ*_1_ (ns) | *A*_2_ | *τ*_2_ (ns) | *τ*_average_ (ns) |
| --- | --- | --- | --- | --- | --- |
| Pristine | 0.67 | 6.09 | 0.36 | 30.36 | 23.76 |
| TPPO | 0.53 | 7.62 | 0.48 | 73.18 | 66.42 |
| 2-F-TPPO | 0.36 | 6.87 | 0.49 | 78.09 | 73.81 |

**A*_i_ is the prefactor of the exponential decay function in *I*(*t*)=*I*_0_+*A*_1_exp(-*t*/*τ*_1_) +*A*_2_exp(-*t*/*τ*_2_), and *τ*_ave_ were calculated by the equation *τ*_ave_=(*A*_1_*τ*_1_^2^+*A*_2_*τ*_2_^2^)/(*A*_1_*τ*_1_+*A*_2_*τ*_2_).
